# Tattooing is Mainly Cultural: A Representative Twin Study of Tattooing Determinants

**DOI:** 10.1007/s10519-025-10215-3

**Published:** 2025-02-01

**Authors:** Signe B. Clemmensen, Jonas Mengel-From, Jaakko Kaprio, Jennifer R. Harris, Henrik Frederiksen, Jacob von Bornemann Hjelmborg

**Affiliations:** 1 Department of Epidemiology, Biostatistics, and Biodemography, Institute of Public Health, University of Southern Denmark, Odense, Denmark; 2 Danish Twin Registry, Institute of Public Health, University of Southern Denmark, Odense, Denmark; 3 Department of Clinical Genetics, Odense University Hospital, Odense, Denmark; 4 Institute for Molecular Medicine Finland FIMM, HiLIFE, University of Helsinki, Helsinki, Finland; 5 Center for Fertility and Health, Norwegian Institute of Public Health, Oslo, Norway; 6 Department of Haematology, Odense University Hospital, Odense, Denmark; 7 Department of Clinical Research, University of Southern Denmark, Odense, Denmark

**Keywords:** Tattoo, Twin study, Familial risk, Shared environment, Heritability

## Abstract

Tattooing has become increasingly common in recent decades, yet little is known regarding factors that influence tattoo behavior. Sources of influence will be important, for instance in aiding studies of long-term health effects. From the population-based Danish Twin Tattoo Cohort established in 2021, the study included 9,173 randomly selected twins born 1920–2004. Among these were 4,790 (52%) responders to a questionnaire on tattooing and lifestyle factors. There were 55% females, 22% were monozygotic twins, and the median age was 51 years. Shared influence of tattooing over time was assessed by comparing monozygotic and dizygotic twin pairs. Responders were population representative on sex, age, and lifestyle factors. The cumulative incidence of being tattooed before age 25 years increased markedly from 6% (95% CI: 4–7%) for males and 0% (0–1%) for females born in 1925–1960 to 30% (25–35%) for males and 41% (37–46%) for females born in 1981–2004. Tattooing was over twice as common among ever smokers compared to never smokers born in 1981–2004 (average smoking effect at age 25 years: 36% (29–43%)). The likelihood of a twin getting tattooed if the co-twin is tattooed, was 2.0 (1.4–2.6) and 1.8 (1.5–2.2) times higher, for monozygotic and dizygotic twins, respectively. The findings indicate that variation in the likelihood of becoming tattooed is primarily explained by shared environmental factors 65% (35–95%), and that genetic influences explained little of this variation. This study demonstrates that strong environmental exposures shared by twin siblings irrespective of degree of genetic relatedness drive the choice for getting tattooed. We conclude that tattooing is a cultural group clustering phenomenon that goes beyond genetically oriented behavioral characteristics.

**Supplementary Information:**

The online version contains supplementary material available at 10.1007/s10519-025-10215-3.

## Introduction

Tattooing is an old tradition and is associated with specific subcultures but has also been a mark of rebellion. In recent decades the popularity of tattooing has increased considerably in Western countries and has moved from subcultures to the mainstream (Kluger 2015). The motivations to get a tattoo are several, e.g., religious or subcultural group affiliation signs, as social status markers or as signs of strength (Wohlrab et al. 2007). This has led to a wide interest in understanding the factors that influence tattooing, including among public health professionals interested in e.g. dermatological (Friis et al. 2024; Klugl et al. 2010) or oncological consequences (Foerster et al. 2020), psychologists interested in the relationship between tattooing with risk-taking (Mortensen et al. 2019) and life-style choices (Carson 2014), and sociological perspectives to study the individual determinants and surrounding cultural influences that affect the choice of body-modifying self-expression. Generally, from a medical point of view, adding foreign substances to one’s body can pose a health risk. With respect to tattoos, the ink is known to accumulate in lymph nodes in the area of the tattoo, which raises questions about immune responses and disease risk. For instance, three studies indicate an increased risk of lymphoma among tattooed individuals (Nielsen et al. 2024; Clemmensen et al. 2024; McCarty et al. 2024) and one study finds that tattoo ink induces inflammation and affects the immune response (Capucetti et al. 2024). Only now as tattooing has become more common, is it possible to assess its public health impact – particularly information on long-term health effects is extremely limited. In that aspect, insights to sources of influence on becoming tattooed will be important and is the main motivation for the present study.

In some European countries, the prevalence of tattooing was about 13–14% in 2011–2015, which increased to 17–21% in 2017–2021 (Bjerre et al. 2018; Renzoni et al. 2018; Institut d’études opinion et marketing en France et à l’international. Les Français et le tatouage 2016; Kluger et al. 2019; Knudsen 2018; Sagoe et al. 2017; Nielsen et al. 2023). There is consistent evidence that tattoos are most popular among the younger generations where the prevalence can be twice the average among other age groups. There is also variation in tattoo popularity between males and females. Generally, in these recent studies females are reported to have a higher tattoo prevalence than males (Sagoe et al. 2017). Studies suggest this difference may be age-dependent, with a higher proportion of young females being tattooed compared to males of similar age (Klugl et al. 2010; Heywood et al. 2012).

Literature focusing on the decision of getting a tattoo is limited. A study from 2002 among American college students found a relationship between tattooing and having tattooed friends and suggested limited familial influence – highest among sisters (Armstrong et al. 2002). The importance of peer influence was also highlighted in a review paper from 2015 (Kluger 2015). In addition to quantifying the changing demographics of tattooing (e.g., sex, age, and generation), there is an interest to understand the underlying factors that influence becoming tattooed.

Cultural factors experienced through belonging to certain groups or communities are influential for tattooing (Armstrong et al. 2002). However, most types of behavior are also partly influenced by heritable differences between people expressed as genetic liability (Dick 2005). Knowledge of the relative importance of genetic and environmental influence on tattooing is lacking. Family and twin designs are valuable for addressing this question.

In twin studies, shared influences of a behavior (such as being tattooed) are indicated by an increased likelihood that a twin will have a certain behavior, given that the same behavior is also present in their co-twin, compared to the prevalence of the behavior in the general population. Further, comparing this conditional likelihood between monozygotic (MZ) and dizygotic (DZ) twins provides robust information on the importance of genetic influence. The variation in probability that cannot be explained by genetic effects is assumed to be due to environmental effects, that can be parsed further into shared (between twin siblings) and non-shared (unique to each twin) effects. The rationale is that if the within-pair similarity is the same among MZ and DZ twins and is elevated in comparison to similarity of individuals, then sources of variation must be factors shared by both twins, which implies shared environmental effects. To our knowledge, there are no prior genetically informative studies or population representative studies of tattoo-related behavior in families or twins.

This study utilizes the largest ever tattoo cohort of twins aiming to provide novel evidence of the main sources of variation in likelihood of becoming tattooed. That is, what are the relative influence of genes, shared and non-shared environments on variation in tattoo likelihood? Additionally, we aim to describe the cumulative incidence of tattooing by various demographic- and lifestyle factors.

## Materials and Methods

The Danish Twin Register (DTR) is the oldest nationwide twin register in the world (Pedersen et al. 2019). It holds more than 175,000 twins, born in 1870–2009. It has complete ascertainment since 1968 where the Danish Civil Registration System was initiated assigning a unique personal identification number to every citizen.

From the population-based Danish Twin Tattoo Cohort established in 2021, the study included 9,173 randomly selected twins from the Danish Twin Register born 1920–2004 (ages 16–101 at time of survey). A full description of the process of setting up the cohort can be found in Clemmensen (2024).

Invitations were distributed in January 2021 using a public digital mailbox including a link to online the SurveyXact webpage (an online tool for questionnaire surveys). However, a small proportion (7%) were unable to be contacted in this manner and were invited through letter mail instead during March 2021. The survey was closed on July 8, 2021, after about a month without new registrations.

The questionnaire included items about tattoo status and, when relevant, further tattoo details such as colors, size (measured in units of the size of the palm of one’s hand), age at first tattoo, and tattoo-related medical issues such visits to the doctor/hospitals stays and swollen lymph nodes. It also inquired about lifestyle factors; smoking (type and duration), physical exercise, alcohol consumption, and education. Age at response was calculated from date of response and birthdate. Sex was provided from register data. Finally, participants could provide open-ended comments. A flowchart of the questions is provided in Appendix 1 in the supplementary material. The full survey is available upon request.

## Statistical Analysis

### Description of Sample

Total counts and percentage or median and interquartile range (IQR) are provided as suitable for characteristics of the cohort by tattoo status, tattooed individuals by sex, and lifestyle factors (smoking, alcohol consumption, and physical activity) and education by tattoo status.

### Representativeness, Cumulative Incidence of Tattooing and Average Exposure Effects

Cumulative incidence functions of age at first tattoo were estimated for males and females by three groups of birth cohorts (oldest: 1925–1960, middle: 1961–1980, and youngest: 1981–2004) using the non-parametric product-limit estimator. Censoring was defined by time of survey participation. From this, one can determine the estimated probability of having at least one tattoo before a specified point in time, e.g., the lifetime probability of having at least one tattoo is defined as the cumulative incidence at age 80 years. Inverse probability sampling weights (IPW) were applied to ensure a distribution of age and sex that was representative of the background population. These weights were determined by the proportion of the number of individuals in each 5-year interval in the background population (Statistikbanken - Danmark Statistik. 2021) to the number of survey participants in corresponding intervals. The age distributions for the cohort and background population are depicted for males in Supplementary Figure S1 and for females Supplementary Figure S2. We further analyzed for sensitivity towards non-participation of co-twin using IPW based on propensity scores from logistic regression (Austin 2011). We also estimated cumulative incidence functions by zygosity.

We estimated the average causal exposure effect (ATE) of sex, smoking, alcohol consumption, and physical activity. That is, if there is no unmeasured confounding we consider the risk difference if everybody were exposed (to e.g. smoking) compared to the situation where none were exposed at the same point in time. For identifying this risk difference in a causal setting, we assume exchangeability, positivity, and consistency (Pearl et al. 2016). We used the binomial competing risk regression model described by Ozenne et al. (Ozenne et al. 2020; Petersen and Lange 2020).

The analysis was performed using two modelling approaches – individual and pair-matched analysis to optimally adjust for (unmeasured) confounding factors. We estimated ATE of having a tattoo before age 25 years among individuals from the youngest generations (1981–2004) for sex, smoking status (defined as ever smoker vs never smoker), alcohol consumption (defined as a weekly intake above vs no higher than 7 standard drinks at follow-up), and physical activity (defined as a weekly level of moderate physical activity above vs no higher than 210 min – corresponding to the recommended 30 min per day – at time of follow-up). The ATE’s were estimated in individual level analysis using binary double robust estimating equations adjusting for censoring by inverse probability censoring weights (IPCW). We further applied IPW for age and sex representativeness in both main and treatment model (weights as defined previously). We adjusted for zygosity in both the main and the treatment model and when modelling the effect of smoking, alcohol consumption and physical activity, we also adjusted for sex in both the main and the treatment model. The pair-matched analysis for improved shared confounder control provided ATE’s by further conditioning on cotwin status of sex or lifestyle factor in both main and treatment model.

These computations were done using the R package *mets* (Holst et al. 2016; Scheike et al. 2014a).

### Familial Dependence

The familial risk, also termed casewise concordance, was defined as the conditional risk of having a tattoo before some age given that the co-twin had a tattoo before that age. The familial risk is given by the ratio of the concordance function, that is, the probability of both twins in a pair getting a tattoo before some age, to the cumulative incidence function. The concordance was estimated using non-parametric counting process modelling as described by Scheike et al. (Scheike et al. 2014b) adjusting for censoring and twin pair clusters and assuming censoring at the same time within twin pairs. Familial risk by age and lifetime familial risk (at age 80 years) was estimated for MZ and DZ twins. An elevated familial risk compared to the individual cumulative incidence is an indication of familial effects – genetic or environmental. Further, assuming environmental influence is independent of zygosity, a higher familial risk among MZ twins compared to DZ twins indicates shared genetic influence. Also, similar familial risks among MZ and DZ twins higher than the individual cumulative incidence indicates shared environmental influence. We analyzed for sensitivity towards non-participation of co-twin using IPW based on propensity scores as described previously.

A direct measure of the relative difference between familial risk and cumulative incidence is given by the relative recurrence risk. Standard errors were obtained through robust variance estimation to account for twin pair-specific similarities. Inference of the measures assume approximate normality in distribution of estimators and test of differences was performed using a Pepe–Mori type test derived similar to the description by Scheike et al. (Scheike et al. 2014b).

Tetrachoric correlations of tattoo status were estimated through bivariate probit modelling, that is, the classic liability-threshold approach, assuming the same cumulative incidence functions for MZ and DZ twins and using inverse probability weighting to account for censoring (Holst et al. 2016; Mucci et al. 2016).

### Biometric Analyses

The overall magnitude of the genetic and environmental contributions to the variation in probability (risk) by age of having a tattoo, taking the timing of events into account, can be obtained via the polygenic biometric model from quantitative genetics, the “ADCE model” (Holst et al. 2016; Sham 1998). The components that contribute to variance are additive genetic (A), dominant genetic (D), shared environmental (C), and unique environmental (E) effects. A biometric model that simultaneously estimates three of the four components can be analyzed using the twin design. In particular, heritability and shared environmentality of probability and also of liability to tattooing (described below) were sought estimated assuming cumulative incidence functions for MZ and DZ twins to be equal (Scheike et al. 2014a). Heritability of probability is defined as the proportion of variation in probability of having a tattoo attributed to genetic factors and is estimated as the ratio of twice the difference in MZ and DZ concordance probability to the total variation in probability (Scheike et al. 2014a; Hjelmborg et al. 2014). Shared environmentality of probability is estimated as the difference between twice the DZ concordance and the MZ concordance relative to the total variation in probability. Additionally, we estimated genetic and environmental contributions to the variation in liability of having a tattoo using the bivariate probit liability-threshold approach (Holst et al. 2016). The most parsimonious sub-model was determined by standard model selection criteria.

The proportion of shared genes between DZ twins is the same as among full siblings, enabling generalization of results on familial dependence.

All analyses were performed using the statistical software *R* version 4.1.0 (R: A language and environment for statistical computing (R Foundation for Statistical Computing,Vienna, Austria 2021). All tests were two-sided and performed at the 5% significance level.

## Results

### Descriptives of Cohort

Among 9,173 invited individual twins, including 3,922 twin pairs, 4,790 (52%) participated, including 1,327 twin pairs (34% of pairs). More females (55%) participated than males (44%) and participation was fairly evenly distributed across 5-year age intervals (see Supplementary Figure S3 for details). The participation rates were 57% among monozygotic twins and 52% among dizygotic twins.

Among the 4,790 individual twin participants in the cohort, 1,061 (22%) had at least one tattoo (Table 1). The median age at participation, which in this case was the same as the median follow-up time, was 38 years among individuals with tattoos and 55 years among those without. There was a slightly larger proportion of females in both groups; 59% of the tattooed twins and 54% of those without tattoos. Among the participants were 73% dizygotic twins. Descriptive information on tattoos, such as size and color, is provided in Supplementary Table S1, and descriptive information on lifestyle factors and education by tattoo status are listed Supplementary Table S2.

**Table 1:** Characteristics of the cohort born 1925–2004 by tattoo status as of 2021

	Tattoo	No Tattoo	Total
Median follow-up time (IQR), years	38.4 (25.4–52.9)	55.1 (31.4–69)	51.1 (28.6–65.6)
Individual twins, n	1,061	3,729	4,790
Females, n (%)	628 (59)	2,026 (54)	2,654 (55)
Zygosity, n (%)			
MZ	224 (21)	834 (22)	1,058
DZ	806 (76)	2,695 (72)	3,501
Unknown	31 (3)	200 (5)	231

*IQR* Interquartile range, *n* Number of observations, *MZ* Monozygotic, *DZ* Dizygotic

Each participant was also asked whether their co-twin had a tattoo. Comparison of self-reported tattoo status and information provided by the co-twin, yielded a high estimated accuracy of 98.5% among the 1,327 pairs where both twins participated.

## Cumulative Incidence of Age at First Tattoo

**Fig. 1:**
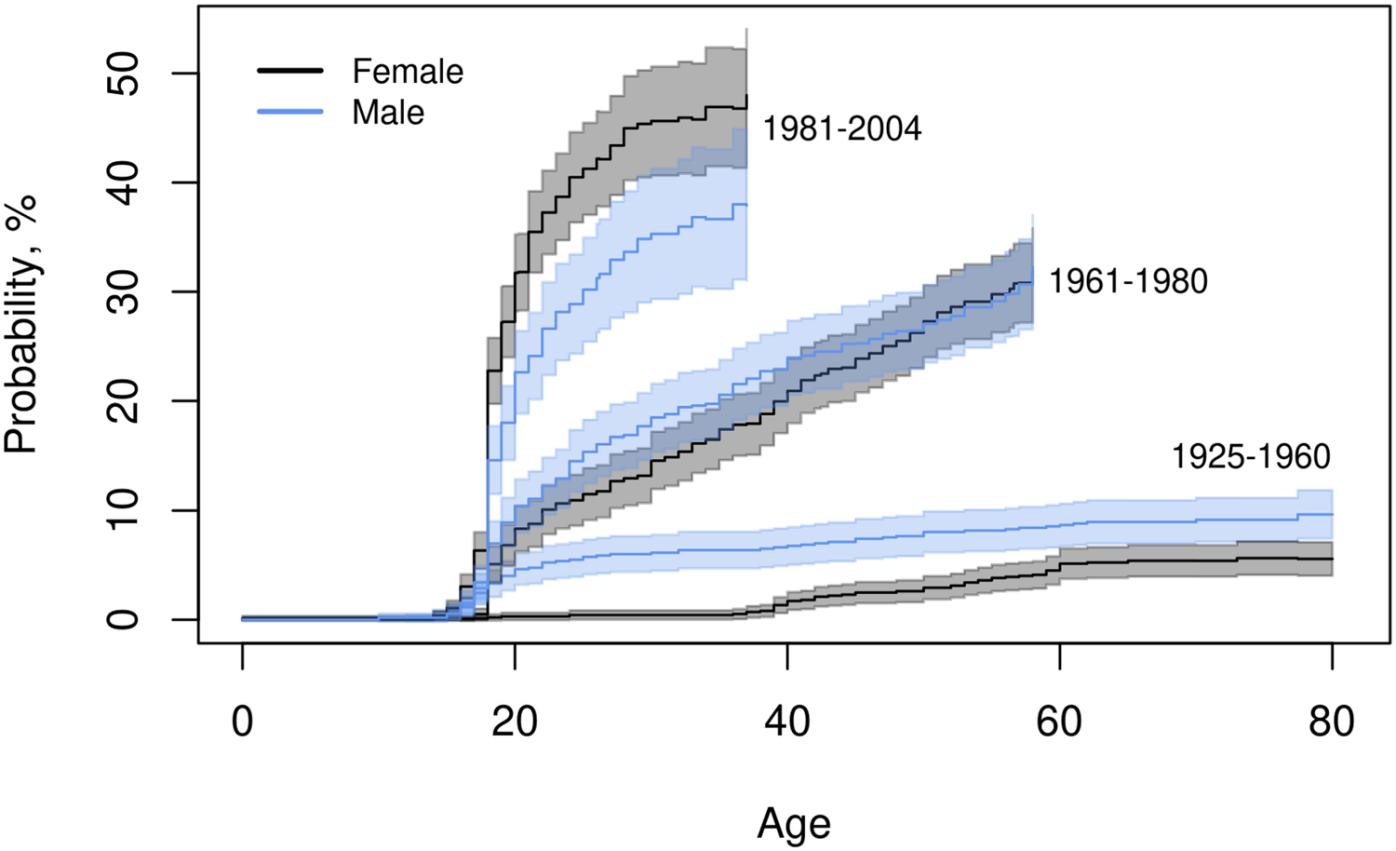
Cumulative incidence by sex and birth cohort. Cumulative incidence and 95% confidence intervals for age at first tattoo by sex and birth cohorts. Inverse probability weight adjustment for population representativeness

The IPW adjusted representative cumulative incidences of age at first tattoo by sex for three groups of birth cohorts are shown in Fig. 1. The cumulative incidence at age 25 years has increased markedly from oldest to youngest birth cohorts. The cumulative incidences were: 5.8% (95% CI: 4.2–7.4%) for males and 0.4% (0.0–0.8%) for females in the oldest group, 15.4% (12.5–18.3%) for males and 11.5% (9.1–13.9%) for females in the middle group, and 30.2% (25.4–35.0%) for males and 41.3% (37.0–45.5%) for females in the youngest group. Further, while tattoos used to be more common among males, that was not the case for those born in 1981–2004. More than half of the tattooed males and females born in 1981–2004 were younger than 20 years old when they had their first tattoo. There was no indication of sensitivity towards non-participation of co-twin in the cumulative incidences.

Stratifying the cumulative incidence function for tattoo exposure by zygosity instead of sex, did not indicate a difference between MZ and DZ individual twins in any of the three groups of birth cohorts. (Supplementary Figure S4).

As there was either no or a small difference between the estimated cumulative incidences with or without IPW adjustment for population representativeness, the unadjusted results are not shown.

## Average Causal Risk Difference (ATE)

We assessed the average causal risk difference in those born in 1981–2004. For smoking behavior and having a tattoo before age 25, ever-smokers (current or former smoker) vs never-smokers showed an average causal risk difference of 35.9% (29.2–42.7%) (Table 2). The absolute risks for ever smokers of 65.2% (59.1–71.3%) was more than double the risk for never-smokers 29.3% (26.4–32.2%). The matched analysis provided similar results with an average causal risk difference of having a tattoo, conditioning on co-twin smoking status, that was 30.3% (19.6–41.0%). Thus, indicating a strong link between smoking and being tattooed even controlling for shared confounders.

**Table 2:** Absolute risk and average causal risk difference (ATE) of having a tattoo before age 25 years by sex and lifestyle factors. Inverse probability weight adjustment for population representativeness

	Individual analysis (%, 95% CI)	Matched analysis ^i^ (%, 95% CI)
Female	42.2 (38.6–45.8)	37.6 (31.7–43.6)
Male	32.3 (28.2–36.5)	32.7 (24.6–40.7)
ATE (sex)	9.9 (4.4–15.4)	5.0 (− 7.0–19.1)
Ever smoker	65.2 (59.1–71.3)	59.8 (50.0–69.7)
Never smoker	29.3 (26.4–32.2)	29.6 (25.5–33.6)
ATE (smoking)	35.9 (29.1–42.7)	30.3 (19.6–41.0)
Alcohol		
> 7 units per week	43.1 (35.0–51.3)	35.1 (24.7–45.4)
≤ 7 units per week	37.2 (34.2–40.2)	35.9 (31.8–40.0)
ATE (alcohol)	5.9 (− 2.8–14.7)	− 0.8 (− 12.0–10.4)
Physical activity ^ii^		
Active	37.0 (31.9–42.0)	33.2 (26.4–40.0)
Inactive	38.8 (35.5–42.1)	38.4 (33.9–42.9)
ATE (phys. activity)	− 1.9 (− 8.0–4.2)	− 5.2 (− 13.4–3.1)

^i)^Among twin pairs discordant for exposure (e.g., sex or smoking)

^ii)^Active defined as more than 210 min of physical activity per week of moderate intensity

The results of the individual analysis further indicated an average causal risk difference for sex that was 9.9% (4.4–15.4%). The absolute risks, 32.3% (28.2–36.5%) for males and 42.2% (38.6–45.8%) for females, were similar to those from the non-parametric cumulative incidence function. The matched analyses yielded no significant differences, this suggests for opposite twin pairs that females having a tattooed brother are as likely to be tattooed as males having a tattooed sister.

No significant average causal risk difference of having a tattoo could be detected for alcohol consumption or physical activity.

## Familial Risk of Tattoo by Age

**Fig. 2:**
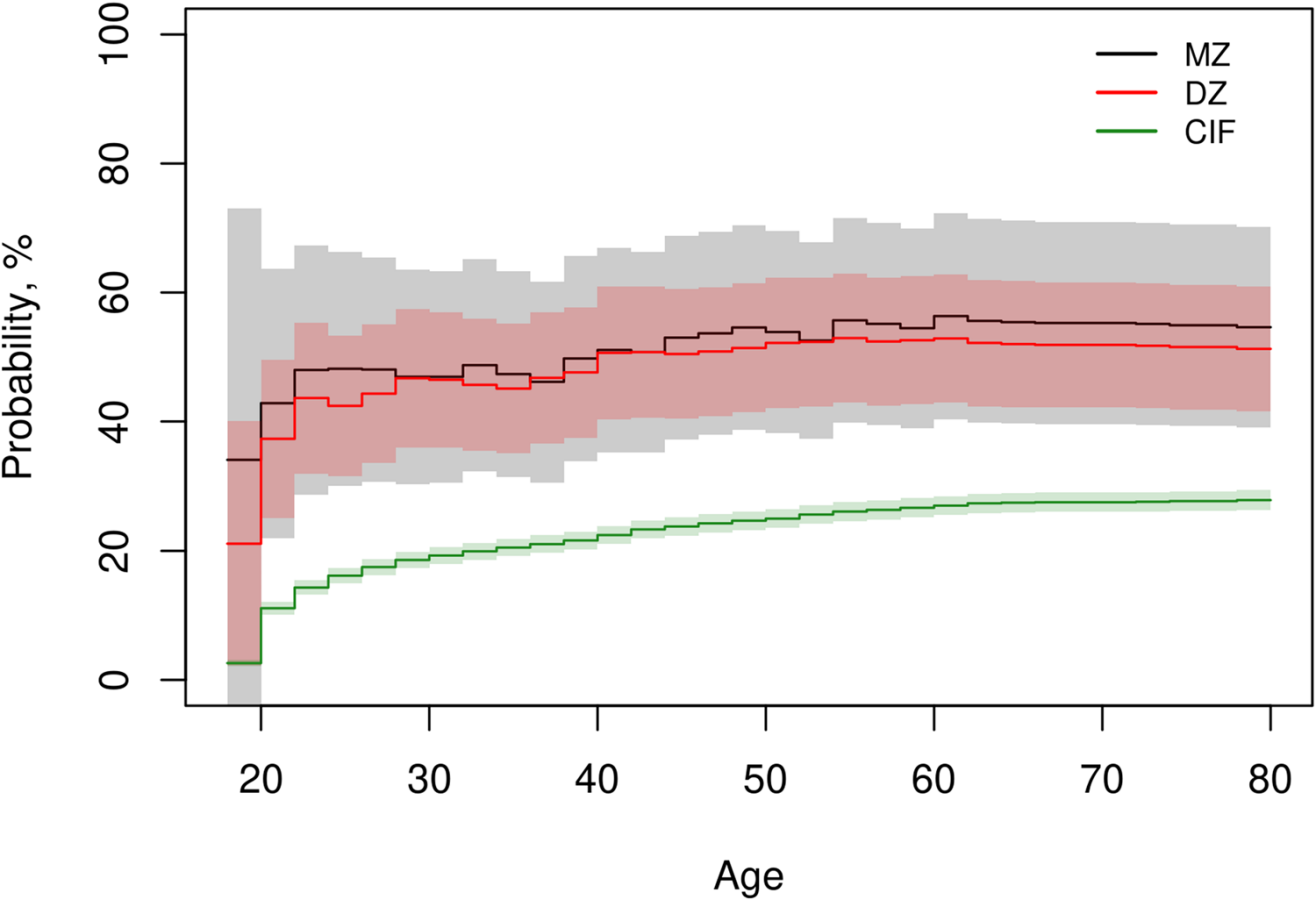
Cumulative incidence and familial risk by zygosity. Cumulative incidence (CIF) and familial risk for monozygotic (MZ) and same sex dizygotic (DZ) twins by age at first tattoo and 95% confidence intervals

Comparing the cumulative incidences to familial risk of age at first tattoo by zygosity for same sex twin pairs, clearly indicated a familial predisposition (Fig. 2). Furthermore, the lack of difference between the MZ and DZ familial risks suggested that this predisposition was primarily explained by shared environmental effects. Stratification by sex suggested differences between males and females (Supplementary Figure S5), but this was not significant. Additionally, MZ males from the oldest birth cohorts tended to be more alike than DZ males, which could indicate some genetic influence, but this was not significant. There was no indication of sensitivity towards non-participation of co-twin in familial risks by age.

A direct measure of the relative difference between familial risk and cumulative incidence was given by the relative recurrence risk (Supplementary Figure S6). It indicated that the likelihood of getting a tattoo, given that the co-twin also had a tattoo, was 2.0 (1.4–2.6) and 1.8 (1.5–2.2) times higher, for MZ and DZ twins, respectively.

The number of pairs without tattoos along with the number of concordant and discordant pairs by zygosity are also provided in Table 3. Only complete pairs where both twins participated in the survey, were included in these counts and in the concordance-related estimates (familial risk, heritability and shared environmentality).

**Table 3:** Estimates of lifetime individual probability and familial risk, tetrachoric correlations, the number of pairs without tattoos, and number of pairs concordant and discordant for having a tattoo

	Lifetime Probability, % (95% CI)^i^	Number of Twin Pairs						Familial Risk, % (95% CI)^i^		Tetrachoric correlations, (95% CI)	
		MZ			DZ^ii^						
		None	Disc	Conc	None	Disc	Conc	MZ	DZ^ii^	MZ	DZ^ii^
Female	28.7 (26.6–30.8)	137	34	27	184	47	44	55.2 (34.9–75.4)	68.4 (48.5–88.5)	0.74 (0.54–0.86)	0.77 (0.62–0.86)
Male	27.4 (25.1–29.7)	102	20	18	137	38	17	59.0 (32.0–86.0)	39.1 (20.7–57.4)	0.81 (0.60–0.92)	0.59 (0.34–0.77)
Overall	28.0 (26.5–29.6)	239	54	45	618	191	108	54.6 (39.1–70.1)	51.3 (41.6–60.9)	0.77 (0.64–0.86)	0.70 (0.57–0.79)

^i)^Estimated at age 80 years. ^ii)^Only same sex twin pairs when stratifying by sex. *CI *Confidence interval, *MZ *Monozygotic, *DZ *Dizygotic

The lifetime familial risks estimated at age 80 years, of having at least one tattoo were: MZ: 54.6% (95% CI: 39.1–70.1%) and DZ: 51.3% (41.6–60.9%). That is, the lifetime probability of having a tattoo was around 50–55% given that the co-twin had a tattoo. Stratification by sex yielded estimates suggesting differences by zygosity, but these were not significant (Table 3). For opposite sex DZ pairs, the familial risk was 48% (35–62%), similar to the overall risk.

Estimates of tetrachoric correlation are also shown in Table 3. Among females, there was no indication of tetrachoric correlations being different for MZ and DZ pairs, whereas the point estimates and 95% confidence intervals indicate greater tetrachoric correlations for MZ than DZ pairs among males.

**Fig. 3:**
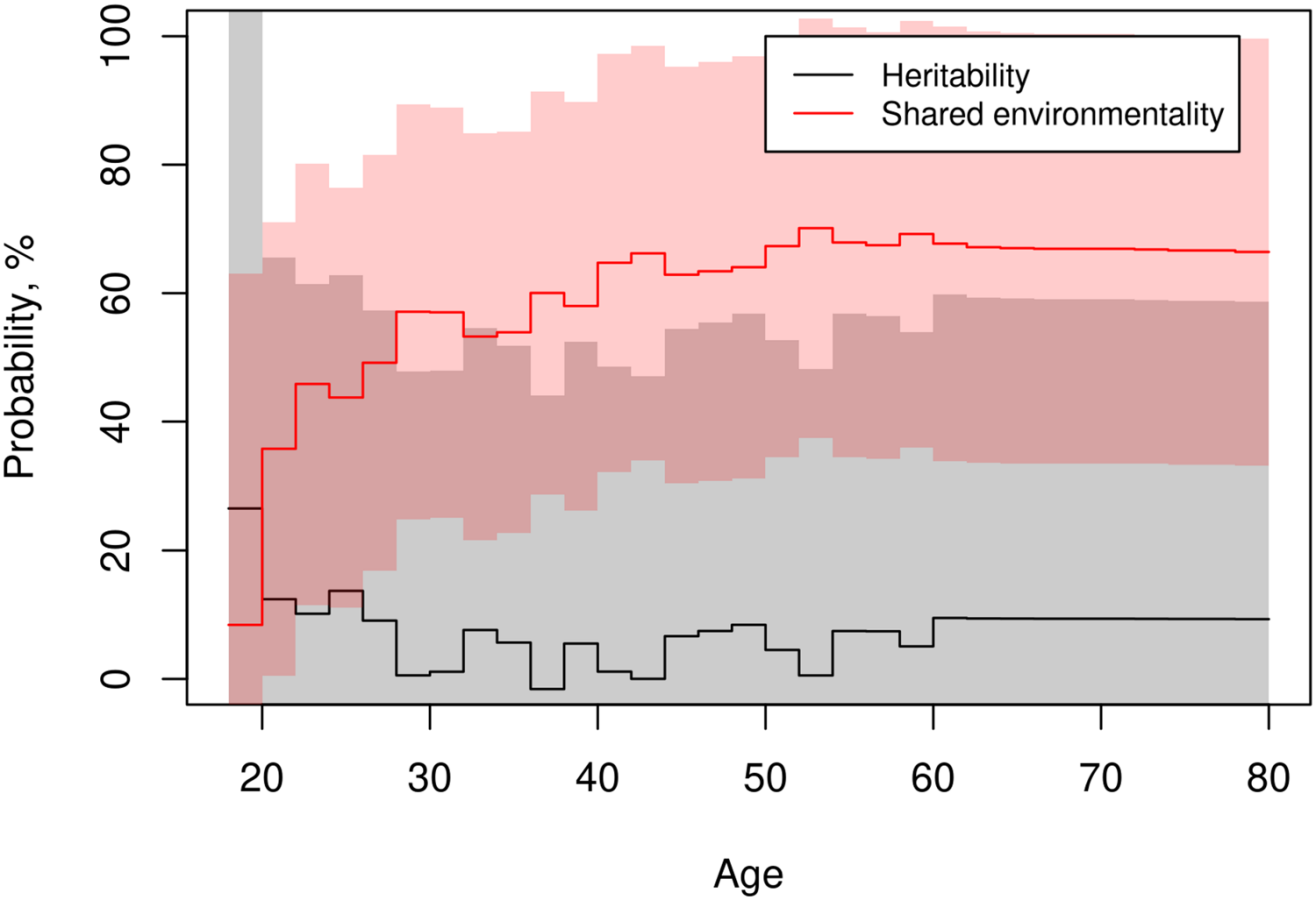
Heritability and shared environmentality. Heritability and shared environmentality of likelihood (probability) of having a tattoo by age (solid lines) and 95% confidence interval (shaded areas)

The heritability and shared environmental contributions to variation in probability of having a tattoo by age are shown in Fig. 3. The shared environmentality appeared to increase with age, to around 65%, though the confidence intervals were rather wide (around 35–95%). The heritability was not significantly different from zero at any age. The remaining variation was accounted for by the unique environmental contribution. Stratification by sex or birth cohort groups was not feasible. We also attempted to estimate heritability and environmentality on the classic liability scale. Through polygenic model selection, the CE-model was chosen as the most parsimonious having the lowest AIC (overview provided in Supplementary Table S3). Shared and unique environmental contributions to variation in liability of having a tattoo by age (only possible up to age 58 years) and 95% confidence intervals are shown in Supplementary Figure S7. Employing an ACE-model resulted in estimates of zero heritability for all ages (figure not shown).

## Discussion

This study lends new insights into the epidemiology and familial effects associated with tattooing. We found: 1) Tattoo cumulative incidence demonstrating the dramatic increase in popularity over the past century. 2) Familial risk of having at least one tattoo is nearly twice as high as the risk in the background population. 3) Sources of variation in probability of being tattooed are primarily due to shared environmental effects. Hence endogenous effects that make individuals more alike are key in explaining tattooing. This includes cultural grouping effects, that is, the influence of a joint cultural setting of groups an individual mainly belongs to. Of particular relevance is peer grouping – a well-studied type of shared environment (not familial) that effects behavior strongly as individuals transition into adolescence (Tuvblad et al. 2011). 4) Evidence of a strong association of having ever smoked on the likelihood of being tattooed among the younger generations. 5) Indication of a sex difference showing a higher cumulative incidence of tattooing in females than in males among the younger generations. However, this is an effect that might be modified by cultural grouping effects (in sense of item 3 above).

The estimated cumulative incidence of tattooing expectedly reflects that of the general population as has been seen for other behaviors after childhood that have been studied in twins (Christensen and McGue 2022). Some of the latest studies on tattooing in European countries have reported survey-based prevalence up to 21% (Sagoe et al. 2017; Nielsen et al. 2023). In this study, we used the cumulative incidence function with IPW adjustment for age and sex population representativeness, which allowed for a more general measure of tattoo occurrence not previously reported. Besides, the stratification by birth cohorts, grouped as old, middle, and young generations, yielded cumulative incidences among the youngest generations (born 1981–2004) suggesting a substantial increase in recent years.

Additionally, we found strong evidence of shared environmental influence for all ages, and very little evidence of genetic influence on the variation in cumulative incidence of being tattooed. Our findings show similar familial risks (cumulative incidences conditioning on co-twin tattoo status) for MZ and DZ twins (55% and 51%), and that this familial risk greatly exceeds the cumulative incidence of 28% in the population. This indicates that shared environmental effects are the main source of variation in cumulative incidence of being tattooed. We presume that these findings will generalize to other Western European countries.

Besides, this characterization of tattooing as a behavior governed by environmental effects with little to no evidence of genetic influence is unusual in behavioral studies. Shared environmental effects are described by behavior geneticists as influences that make siblings more similar to one another and, typically, the impact of shared environment decreases with increasing age as siblings become more independent of each other (Dick 2005). Classic examples of shared environmental influences are childhood factors (e.g. household, parents, peers), but there are other potential factors such as shared acquaintances later in life and common exposures external to the individual family, e.g., social media, awareness campaigns (by government or health organizations) or events influencing larger groups (e.g. pandemics or war). Besides, strong assortative mating can mimic shared environmental effects. While tattoos themselves were rarer in parental generations, obvious phenotypic assortment on having a tattoo is an unlikely explanation. However, behavioral characteristics such as psychological susceptibility to cultural innovations, perhaps even novelty seeking or more generally externalizing behaviors (which associate also with smoking, an important covariate of tattoo behavior), may be present in parental generations.

While we are the first, to our knowledge, to use twins to assess shared genetic and environmental influences on having a tattoo, previous studies have focused on the role of ordinary siblings and parents. In line with our finding of shared environmental influence, Kluger (Kluger 2015) points out that peer influence has been shown to play an important role in getting a tattoo. Armstrong et al. (Armstrong et al. 2002) found being tattooed significantly related to having friends with tattoos, but family only had limited influence – the only significant effect (p = 0.05) found among sisters.

Previous studies have suggested modification of prevalence by age, with a higher proportion of young females being tattooed compared to males of similar age (Sagoe et al. 2017; Heywood et al. 2012). This was not directly tested in our study, but it is consistent with tattoos being more common among males in the oldest birth cohorts and more common among females in the youngest birth cohorts. Based on a review of studies from different countries, Kluger reports increased smoking among tattooed individuals (Kluger 2015) which also corresponds to our results.

We considered age, education, and sex to be the most likely sources of bias in our study. We have mimicked population representativeness through IPW adjustment. One could use a similar procedure to assess and account for varying participation rates by level of education. That was not done in this study since the education information provided through the survey could not be mapped directly to information obtained through Statistics Denmark (Statistikbanken - Danmark Statistik. Educational attainment (15–69 years) by region, ancestry, highest education completed, sex, age and time. 2021). A rough comparison of age distributions by sex for participants and the background population for different levels of education in 2021 suggested higher participation rates among those with higher levels of education which could bias our data towards lower tattooing rates according to previous findings (Kluger 2015; Sagoe et al. 2017). Furthermore, age distributions by sex for smoking status and alcohol consumption were found to be very similar to results from a national health survey with more than 170,000 participants (Jensen et al. 2021). However, the national survey was reportedly prone to similar participation bias as described previously.

For familiar risk curves in Fig. 2, we infer that the curves are really the same as we have no evidence against the null hypothesis that they are equal at all time points. This because the curves do not deviate by more than plus minus 5% and do intertwine during the entire age interval until at least age 60.

Another potential source of bias, which could not be assessed, relates to the main topic of the survey – tattoos. While the title of the survey (*Risk factors of certain types of cancer diseases*) did not refer to tattoos, the invitation mentioned tattoos in the explanation of the project aims. It is possible that some based their decision of whether to participate on their personal opinion on tattoos or thinking it was related to cancer. Additionally, several inquiries made by participants questioned why they received such a survey and whether their contribution would be relevant for the study, since they did not have any tattoos. Furthermore, there are several different types of tattoos. The intended focus of this study was “classic” decorative tattoos. However, since the survey did not specify type of tattoo to the participants, individuals with permanent make-up (PMU) and medical tattoos may also have responded as being tattooed. This may have resulted in misclassification in the tattoo variable. The survey was executed during a period of COVID-19 national lockdown. Several of the participants commented that their reported levels of physical exercise and alcohol consumption deviated from before the lockdown and should therefore be treated with consideration.

A weakness of the matched analysis in this study is that it required survey participation from both twins in a pair which was not always possible. Besides, zygosity was unknown for a small fraction of the twins born in 2001–2004, which meant they had to be excluded from the analyses of shared influence. This can influence the accuracy of the inference, but not the risk estimates themselves.

Timing is a limitation of this study when it comes to association between tattooing and lifestyle factors, especially alcohol consumption and physical activity because the age at first tattoo and age at which the questionnaire was answered (where information on lifestyle factors was provided) may vary up to several decades. This was not considered a problem with smoking because we looked at ever vs never smokers and a majority of the smokers started smoking at young ages (median (IQR): 16 years (15–18 years)). While smokers were more represented among tattooed twins, the possible association might be very complex. Insights to the question of common factors underlying smoking habits and tattooing can be pursued by the twin design using the multivariate biometric twin model (Bonat and Hjelmborg 2022).

Generally, from a medical point of view, adding foreign substances to one’s body is a health risk a priori. Only now as tattooing has become more common, is it possible to assess its public health impact. We hope that the present study will aid in such endeavors. In particular, that the empirical evidence describing how common tattooing has become will hopefully emphasize the need for public health studies – especially the potential long-term health effects of tattooing. Besides, information on sources of influence on becoming tattooed will be important for public health strategies in such cases. For instance, among instances of public health related issues could be tattoo ink induced carcinogenicity. Through linkage with the Danish National Cancer Registry, this cohort, along with the case-cotwin study of around 1,300 twins, has further potential to assess the severe lack of empirical evidence pertaining to carcinogenicity of tattoo ink.

In conclusion, we have shown empirically that tattooing has increased in popularity over the past century and that it is a cultural phenomenon with little to no evidence for genetic influences. With the seemingly still increasing popularity of tattooing, further effort should be made to follow tattooed persons for potential short and long-term effects of being tattooed taking the associated factors such as age and smoking into accounts.

## Supplementary Information

Below is the link to the electronic supplementary material.

Supplementary file1 (PDF 760 KB)

## Data Availability

Restrictions apply to the availability of these data. Requests to access data need to be made through the Danish Twin Registry.

## References

[CR1] Armstrong ML, Owen DC, Roberts AE, Koch JR (2002) College students and tattoos. Influence of image, identity, family, and friends. J Psychosoc Nurs Mental Health Serv. 10.3928/0279-3695-20021001-07 10.3928/0279-3695-20021001-07 12385196

[CR2] Austin PC (2011) An introduction to propensity score methods for reducing the effects of confounding in observational studies. Multivariate Behav Res 46:399–424. 10.1080/00273171.2011.568786 21818162 10.1080/00273171.2011.568786 PMC3144483

[CR3] Bjerre RD, Ulrich NH, Linneberg A, Duus Johansen J (2018) Adverse reactions to tattoos in the general population of Denmark. J Am Acad Dermatol 79:770–772. 10.1016/j.jaad.2018.03.038 29614242 10.1016/j.jaad.2018.03.038

[CR4] Bonat WH, Hjelmborg JVB (2022) Multivariate generalized linear models for twin and family data. Behav Genet 52:123–140. 10.1007/s10519-021-10095-3 35034249 10.1007/s10519-021-10095-3

[CR5] Capucetti, A., Falivene, J., Pizzichetti, C., Latino, I., Mazzuchelli, L., Schacht, V., et al. (2025). *Preprint at bioRxiv*, 2024.12.18.629172 10.1101/2024.12.18.629172

[CR6] Carson HJ (2014) The medium, not the message. How tattoos correlate with early mortality. Am J Clin Pathol 142:99–103. 10.1309/ajcp32fwqlueo 24926092 10.1309/AJCPDOI32FWQLUEO

[CR7] Christensen, K. & McGue, M. in *Twin Research for Everyone* (eds Adam Tarnoki, David Tarnoki, Jennifer Harris, & Nancy Segal) 439–456 (Academic Press, 2022).

[CR8] Clemmensen, S. B. Familial Risk of Hematologic Malignancies – a Twin Study [PhD Thesis]: University of Southern Denmark (2024). 10.21996/day7-6z07

[CR9] Clemmensen, S. B. Mengel-From, J. Kaprio, J. Frederiksen, H. Hjelmborg, J. vB. (2024) Tattoo ink exposure is associated with lymphoma and skin cancers – a Danish study of twins. BMC Public Health 25:170. 10.1186/s12889-025-21413-3 10.1186/s12889-025-21413-3 PMC11736920 39819495

[CR10] Dick, D. M. in *Encyclopedia of Statistics in Behavioral Science* Vol. Vol. 4. (eds Brian S. Everitt & David C. Howell) 1828–1830 (2005).

[CR11] Foerster M, Schreiver I, Luch A, Schuz J (2020) Tattoo inks and cancer. Cancer Epidemiol 65:101655. 10.1016/j.canep.2019.101655 31836426 10.1016/j.canep.2019.101655

[CR12] Friis K, Thomsen AML, Olsen J, Rørth MR, Serup J (2024) Tattoo-associated skin reactions: a danish population-based survey in 5,914 tattooed individuals. Dermatology 240:297–303. 10.1159/000535536 38081147 10.1159/000535536

[CR13] Heywood W, Patrick K, Smith AM, Simpson JM, Pitts MK, Richters J, Shelley JM, Heywood W et al (2012) Who gets tattoos? Demographic and behavioral correlates of ever being tattooed in a representative sample of men and women. Ann Epidemiol 22:51–56. 10.1016/j.annepidem.2011.10.005 22153289 10.1016/j.annepidem.2011.10.005

[CR14] Hjelmborg JB, Scheike T, Holst K, Skytthe A, Penney KL, Graff RE et al (2014) The heritability of prostate cancer in the Nordic Twin Study of Cancer. Cancer Epidemiol Biomarkers Prev 23:2303–2310. 10.1158/1055-9965.EPI-13-0568 24812039 10.1158/1055-9965.EPI-13-0568 PMC4221420

[CR15] Holst KK, Scheike TH, Hjelmborg JB (2016) The liability threshold model for censored twin data. Comput Statis Data Anal 93:324–335. 10.1016/j.csda.2015.01.014

[CR16] Institut d’études opinion et marketing en France et à l’international. *Les Français et le tatouage*, https://www.ifop.com/publication/les-francais-et-le-tatouage/ (2016).

[CR17] Jensen, H. A. R., Davidsen, M., Møller, S. R., Román, J. E. I., Kragelund, K., Christensen, A. I. & Ekholm, O. Danskernes Sundhed - Den Nationale Sundhedsprofil 2021. (Sundhedsstyrelsen, 2022).

[CR18] Kluger N (2015) Epidemiology of tattoos in industrialized countries. Curr Probl Dermatol 48:6–20. 10.1159/000369175 25833619 10.1159/000369175

[CR19] Kluger N, Misery L, Seite S, Taieb C (2019) Tattooing: A national survey in the general population of France. J Am Acad Dermatol 81:607–610. 10.1016/j.jaad.2018.10.059 30395921 10.1016/j.jaad.2018.10.059

[CR20] Klugl I, Hiller KA, Landthaler M, Baumler W (2010) Incidence of health problems associated with tattooed skin a nation-wide survey in German-speaking countries. Dermatology 221:43–50. 10.1159/000292627 20215724 10.1159/000292627

[CR21] Knudsen, H. Vi ved ikke, hvilket blæk vi bliver tatoveret med, https://samvirke.dk/artikler/vi-ved-ikke-hvilket-blaek-vi-bliver-tatoveret-med (2018).

[CR22] McCarty RD, Trabert B, Kriebel D, Millar MM, Birmann BM, Grieshober L et al (2024) Tattoos and risk of hematologic cancer: a population-based case-control study in Utah. Cancer Med 13(20):e70260. 10.1002/cam4.70260 39444249 10.1002/cam4.70260 PMC11499570

[CR23] Mortensen K, French MT, Timming AR (2019) Are tattoos associated with negative health-related outcomes and risky behaviors? Int J Dermatol 58:816–824. 10.1111/ijd.14372 30677140 10.1111/ijd.14372

[CR24] Mucci LA, Hjelmborg JB, Harris JR, Czene K, Havelick DJ, Scheike T et al (2016) Familial risk and heritability of cancer among twins in nordic countries. JAMA 315:68–76. 10.1001/jama.2015.17703 26746459 10.1001/jama.2015.17703 PMC5498110

[CR25] Nielsen C, Andreasson K, Olsson H, Engfeldt M, Joud A (2023) Cohort profile The Swedish tattoo and body modifications cohort (TABOO). BMJ Open 13:e069664. 10.1136/bmjopen-2022-069664 37142309 10.1136/bmjopen-2022-069664 PMC10163470

[CR26] Nielsen C, Jerkeman M, Joud AS (2024) Tattoos as a risk factor for malignant lymphoma: a population-based case-control study. EClinicalMedicine 72:102649. 10.1016/j.eclinm.2024.102649 38827888 10.1016/j.eclinm.2024.102649 PMC11141277

[CR27] Ozenne BMH, Scheike TH, Stærk L, Gerds TA (2020) On the estimation of average treatment effects with right-censored time to event outcome and competing risks. Biom J 62:751–763. 10.1002/bimj.201800298 32049385 10.1002/bimj.201800298

[CR28] Pearl, J., Glymour, M. & Jewell, N. P. Causal Inference in Statistics: A Primer (John Wiley & Sons Ltd Chichester, West Sussex, UK, 2016).

[CR29] Pedersen DA, Larsen LA, Nygaard M, Mengel-From J, McGue M, Dalgard C et al (2019) The Danish twin registry: an updated overview. Twin Res Hum Genet 22:499–507. 10.1017/thg.2019.72 31544734 10.1017/thg.2019.72 PMC8039015

[CR30] Petersen AH, Lange T (2020) What is the causal interpretation of sibling comparison designs? Epidemiology 31:75–81. 10.1097/ede.0000000000001108 31651661 10.1097/EDE.0000000000001108

[CR31] R: A language and environment for statistical computing (R Foundation for Statistical Computing, Vienna, Austria, 2021).

[CR32] Renzoni A, Pirrera A, Novello F, Lepri A, Cammarata P, Tarantino C et al (2018) The tattooed population in Italy: a national survey on demography, characteristics and perception of health risks. Ann Ist Super Sanita 54:126–136. 10.4415/ANN_18_02_08 29916417 10.4415/ANN_18_02_08

[CR33] Sagoe D, Pallesen S, Andreassen CS (2017) Prevalence and correlates of tattooing in Norway: A large-scale cross-sectional study. Scand J Psychol 58:562–570. 10.1111/sjop.12399 29105125 10.1111/sjop.12399

[CR34] Scheike TH, Holst KK, Hjelmborg JB (2014a) Estimating heritability for cause specific mortality based on twin studies. Lifetime Data Anal 20:210–233. 10.1007/s10985-013-9244-x 23378036 10.1007/s10985-013-9244-x

[CR35] Scheike TH, Holst KK, Hjelmborg JB (2014b) Estimating twin concordance for bivariate competing risks twin data. Stat Med 33:1193–1204. 10.1002/sim.6016 24132877 10.1002/sim.6016

[CR36] Sham, P. *Statistics in Human Genetics*. (Arnold, 1998).

[CR37] Statistikbanken - Danmark Statistik. Folketal 1. januar 2021. https://extranet.dst.dk/pyramide/pyramide.htm#!y=2021&v=2&g (2021).

[CR38] Statistikbanken - Danmark Statistik. Educational attainment (15–69 years) by region, ancestry, highest education completed, sex, age and time. https://www.statbank.dk/20017 (2021).

[CR39] Tuvblad C, Narusyte J, Grann M, Sarnecki J, Lichtenstein P (2011) The genetic and environmental etiology of antisocial behavior from childhood to emerging adulthood. Behav Genet 41:629–640. 10.1007/s10519-011-9463-4 21431322 10.1007/s10519-011-9463-4

[CR40] Wohlrab S, Stahl J, Kappeler PM (2007) Modifying the body: motivations for getting tattooed and pierced. Body Image 4:87–95. 10.1016/j.bodyim.2006.12.001 18089255 10.1016/j.bodyim.2006.12.001

