# Supplementary material for: Tattooing is Mainly Cultural: A Representative Twin Study of Tattooing Determinants
Source: Behav Genet. 2025 Feb 1;55(2):114–23. doi: 10.1007/s10519-025-10215-3 (PMC11882612; doi:10.1007/s10519-025-10215-3)
Supplement: Supplementary file 1 — Supplementary file1 (PDF 760 KB) [file 10519_2025_10215_MOESM1_ESM.pdf]

Online Resources 1

Supplementary material

Title: Tattooing is mainly cultural. A representative twin study of tattooing determinants.

Journal: Behavior Genetics

Signe B. Clemmensen[1,2,*] (https://orcid.org/0000-0001-9871-149X), Jonas Mengel-From[1,2,3] (https://orcid.org/0000-0003-1573-8908), Jaakko Kaprio[4] (https://orcid.org/0000-0002-3716-2455), Jennifer R. Harris[5], Henrik Frederiksen[6,7] (https://orcid.org/0000-0001-8905-0220, Jacob vB. Hjelmborg[1,2] (https://orcid.org/0000-0001-9630-9149).

[1] Department of Epidemiology, Biostatistics, and Biodemography, Institute of Public Health, University of Southern Denmark, Odense, Denmark.
[2] Danish Twin Registry, Institute of Public Health, University of Southern Denmark, Odense, Denmark.
[3] Department of Clinical Genetics, Odense University Hospital, Odense, Denmark.
[4] Institute for Molecular Medicine Finland FIMM, HiLIFE, University of Helsinki, Helsinki, Finland.
[5] Center for Fertility and Health, Norwegian Institute of Public Health, Oslo, Norway.
[6] Department of Haematology, Odense University Hospital, Odense, Denmark.
[7] Department of Clinical Research, University of Southern Denmark, Odense, Denmark.

* Corresponding author: sbclemmensen@health.sdu.dk

Appendix 1: Questionnaire overview in English

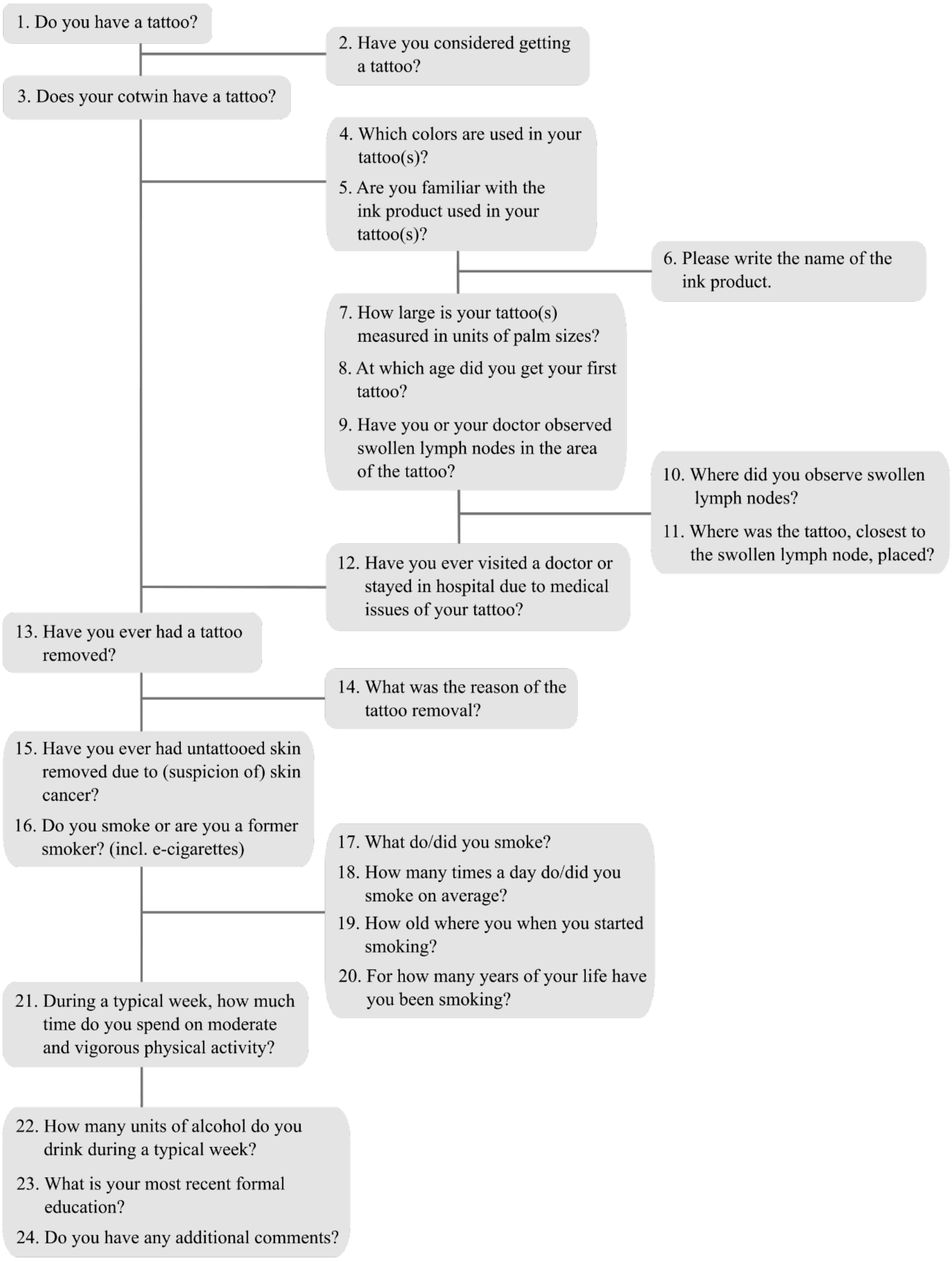

Appendix 2: Age distribution among survey participants

Males

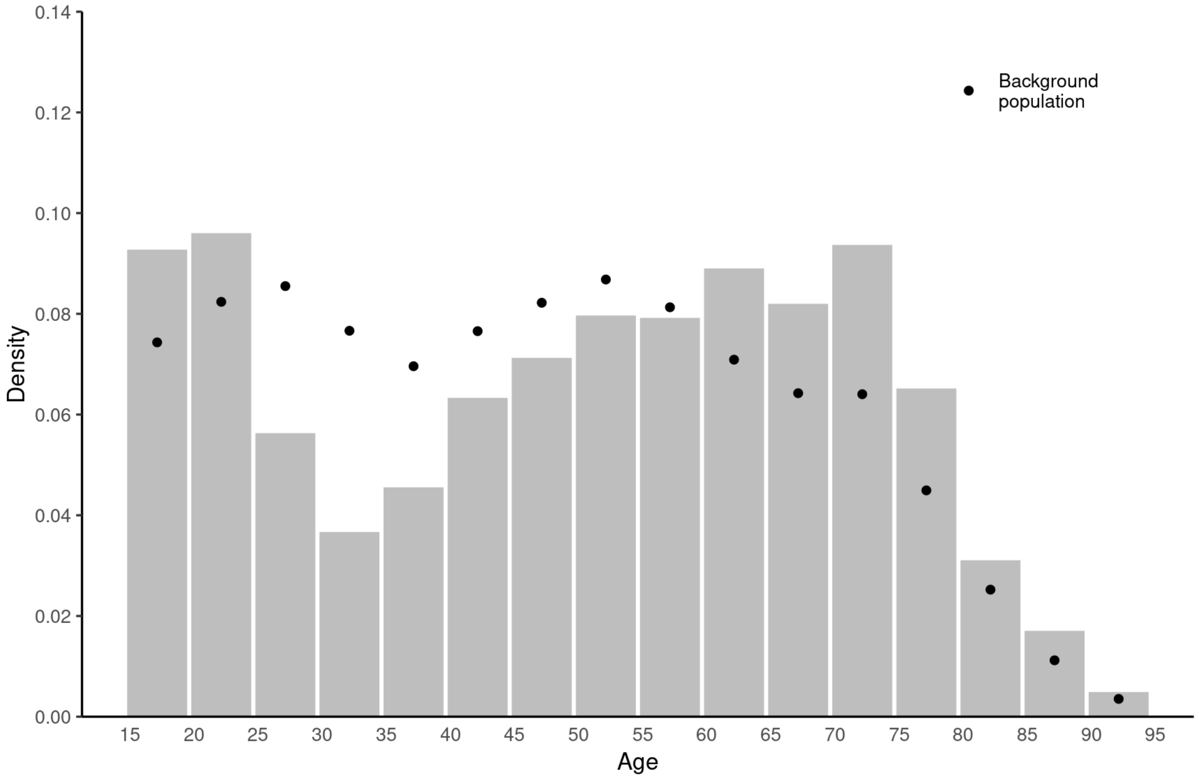

Supplementary Figure S1. Age distribution in cohort and background population among males. Age distribution among the 2,136 male participants in the cohort compared to that of the background population, i.e., the Danish male population above age 15 [19]. Age intervals are half open intervals, e.g. (15-20].

Females

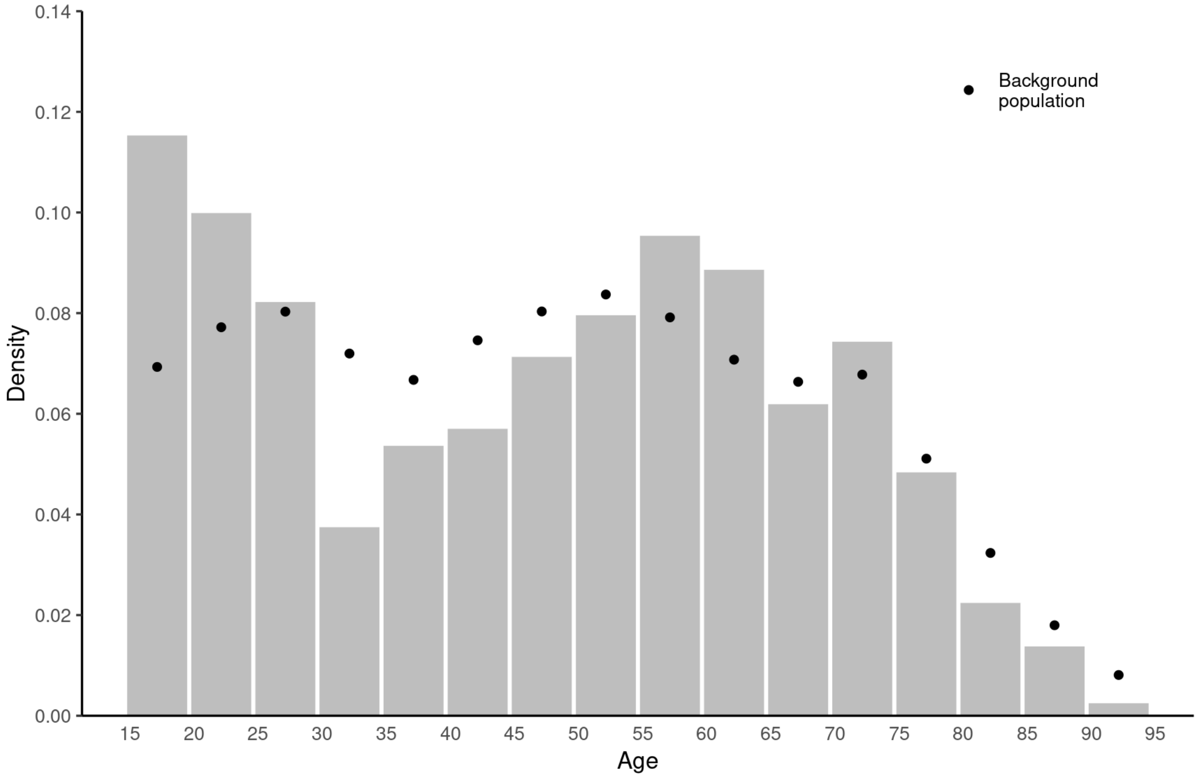

Supplementary Figure S2. **Age distribution in cohort and background population among females**. Age distribution among the 2,654 female participants in the cohort compared to that of the background population, i.e., the Danish female population above age 15 [19]. Age intervals are half open intervals, e.g. (15-20].

Appendix 3: Age distribution among invited twins

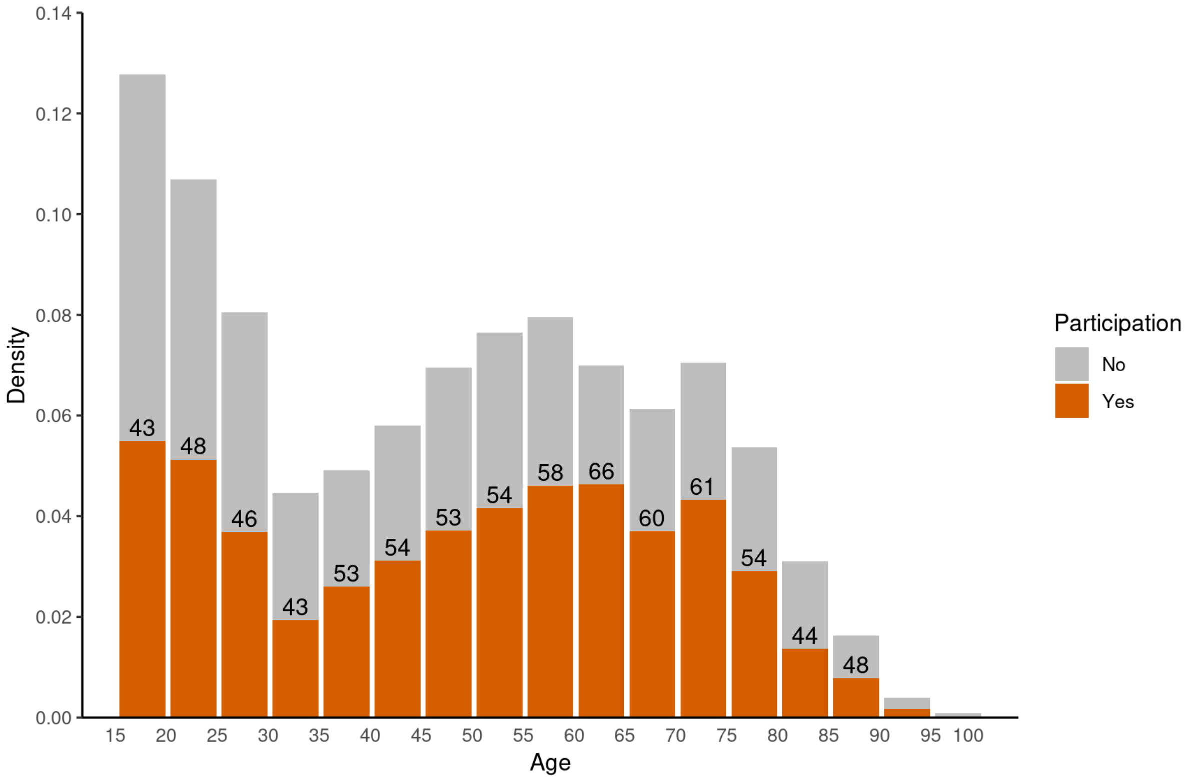

Supplementary Figure S3. Age distribution of invited individual and participation rates. Age distribution among 9,173 invited individuals. The values written above the orange bars indicate participation rates in percentages. Age intervals are half open intervals, e.g. (15-20].

Appendix 4: Descriptives

	Male	Female	Total
Tattoos, n (%)	433 (41)	628 (59)	1,061
Age at first tattoo, years, n (%)			
<20	203 (47)	300 (48)	503 (47)
20-30	140 (32)	159 (25)	299 (28)
31-40	41 (9)	66 (11)	107 (10)
41-50	27 (6)	56 (9)	83 (8)
>50	22 (5)	47 (7)	69(7)
Tattoo colors, n (%) [i]			
Black	393 (91)	590 (94)	983 (93)
Red	132 (30)	119 (19)	251 (24)
Blue	98 (23)	105 (17)	203 (19)
Green	87 (20)	93 (15)	180 (17)
White	67 (15)	84 (13)	151 (14)
Yellow	80 (18)	58 (9)	138 (13)
Grey	29 (7)	39 (6)	68 (6)
Purple	19 (4)	44 (7)	63 (6)
Orange	30 (7)	32 (5)	62 (6)
Other colors	22 (5)	37 (6)	59 (6)
Ink product known, n (%)	11 (3)	14 (2)	25 (2)
Tattoo size measured in units of the palm of one's hand, n (%)			
<1	199 (46)	389 (62)	588 (55)
1-5	166 (38)	193 (31)	359 (34)
>5	65 (15)	41 (7)	106 (10)
Ever needed medical attention in relation to tattoo, n (%)	9 (2)	10 (2)	19 (2)

[i)] Multiple choices allowed.

Supplementary Table S1: Characteristics of tattooed individuals by sex.

Note. n=number of observations.

Only 2% of the participants reported that they knew the name of the ink brand and less than half of these actually provided the name.

Among the questions related to medical attention, two percent of the participants reported having had need for medical attention in relation to their tattoo, and only seven participants reported swollen lymph nodes. Also, 70 (7%) of the tattooed individuals and 409 (11%) of those without tattoos reported having had untattooed skin removed due to (suspicion of) skin cancer.

Among participants without tattoos, 405 (11%) replied that they had considered getting a tattoo. All participants were asked if they had ever had a tattoo removed. Affirmative responses were given by 33 (3%) tattooed individuals and less than five individuals without tattoos.

	Tattoo	No Tattoo	Total
Individual twins, n	1,061	3,729	4,790
Smoking, n (%)			
Current	294 (28)	406 (11)	700 (15)
Former	255 (24)	745 (20)	1,000 (21)
No	504 (48)	2565 (69)	3,069 (64)
Missing	8 (1)	13 (0)	21 (0)
Physical exercise, min/week, median (IQR)			
Moderate	120 (30-210)	108 (30-240)	120 (30-240)
Intense	30 (0-120)	20 (0-90)	30 (0-100)
Missing, n (%)	25 (2)	169 (5)	194 (4)
Alcohol consumption, weekly drinks, n (%)			
0	412 (39)	1,099 (29)	1,511 (32)
1-7	440 (41)	1,707 (46)	2,147 (45)
8-14	107 (10)	487 (13)	594 (12)
15-21	44 (4)	181 (5)	225 (5)
>21	20 (2)	57 (2)	77 (2)
Missing	38 (4)	198 (5)	236 (5)
Education, n (%)			
No formal education	15 (1)	82 (2)	97 (2)
Elementary and middle school	114 (11)	540 (14)	654 (14)
Highschool	162 (15)	309 (8)	471 (10)
Vocational education	305 (29)	761 (20)	1,066 (22)
Degree programmes, 2-2.5 years	92 (9)	243 (7)	335 (7)
Degree programmes, 3-4 years	211 (20)	855 (23)	1,066 (22)
Degree programmes, 5-6 years	65 (6)	516 (14)	581 (12)
Other	65 (6)	249 (7)	314 (7)
Missing	32 (3)	174 (5)	206 (4)

Supplementary Table S2. Distribution of lifestyle factors and education by tattoo status.

Note. n=number of observations, IQR=Interquartile range.

Appendix 5: Results of statistical analysis

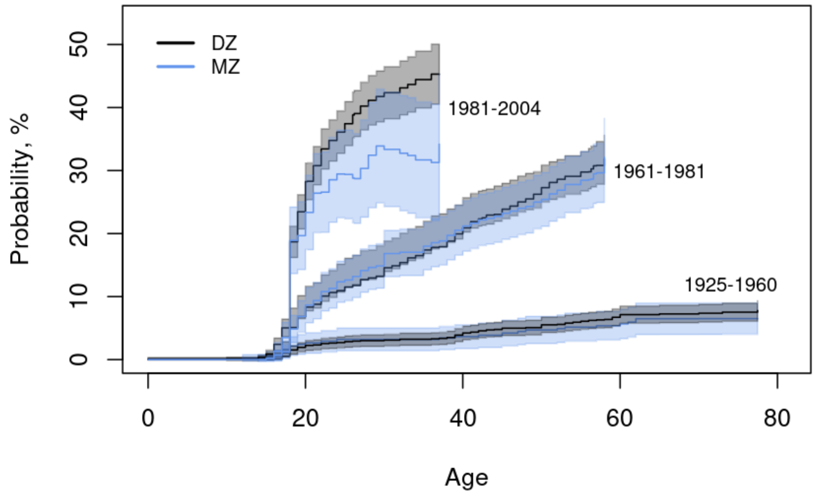

Supplementary Figure S4. **Inverse probability weights adjusted cumulative incidence by sex and birth cohort**. Cumulative incidence and 95% confidence intervals for age at first tattoo by zygosity and birth cohorts. Inverse probability weight adjustment for population representativeness.

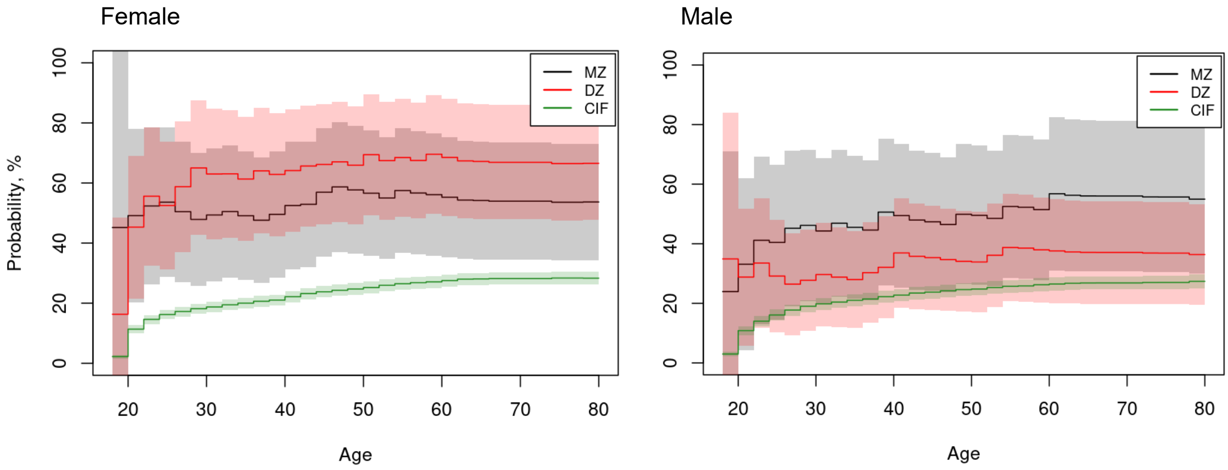

Supplementary Figure S5. **Cumulative incidence and familial risk by zygosity for males and females.** Cumulative incidence (CIF) and familial risk for monozygotic (MZ) and same sex dizygotic (DZ) twins by age at first tattoo and 95% confidence intervals for females and males.

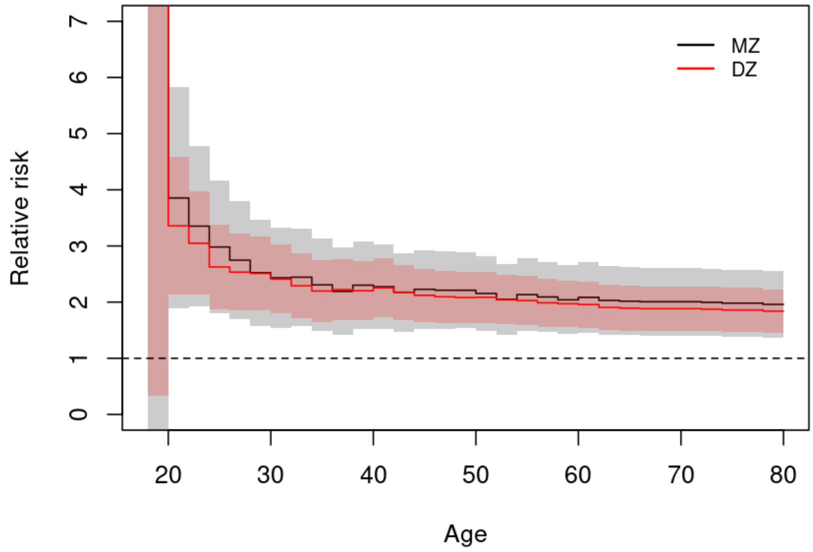

Supplementary Figure S6. Inverse probability weights adjusted relative recurrence risk by zygosity. Relative recurrence risk for monozygotic (MZ) and dizygotic (DZ) twins by age at first tattoo and 95% confidence intervals. Inverse probability weights adjustment for population representativeness.

Model	A (95% CI)	D (95% CI)	C (95% CI)	E (95% CI)	log.lik.	df	p*	AIC
Flex					-2345.406	8		4706.812
U					-2346.184	6	0.4594	4704.368
ADE	0.83 (0.74-0.92)	0 (0-0)	-	0.17 (0.08-0.26)	-2358.702	6		4729.404
ACE	0 (0-0)	-	0.76 (0.67-0.85)	0.24 (0.15-0.33)	-2346.215	6		4704.431
AE	0.83 (0.74-0.92)	-	-	0.17 (0.08-0.26)	-2358.702	4	<0.0001	4725.404
CE	-	-	0.76 (0.67-0.85)	0.24 (0.15-0.33)	-2347.473	4	0.2843	4702.946

* Comparing U to flex and AE and CE to ACE.

Supplementary Table S3. Overview of the polygenic model selection process.

Note. The flex model assumes equal margins within twin pairs, the U model further assumes equal margins for monozygotic and dizygotic twins and the remaining are biometric sub models of the ADCE model for additive (D) and dominant (D) genetic effects and shared (C) and non-shared (E) environmental effects. log.lig.=log-likelihood. df=degrees of freedom, p=p-value, AIC=Akaike's information criterion.

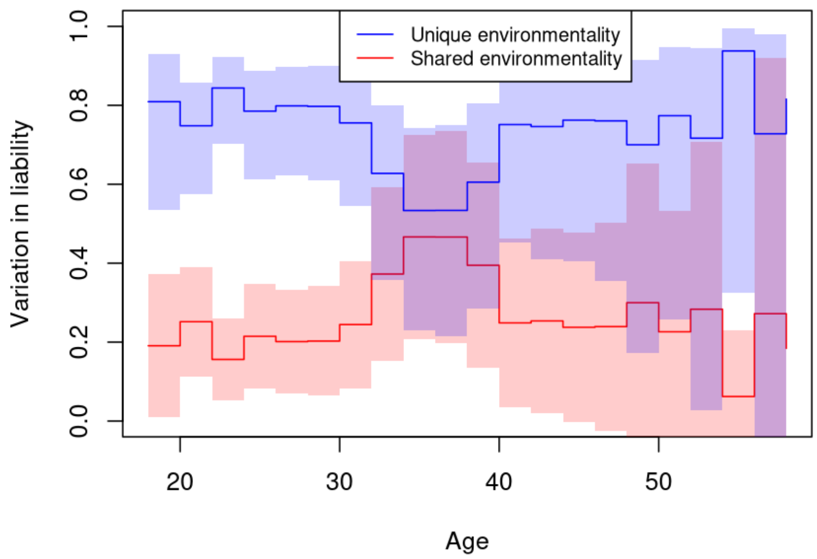

Supplementary Figure S7. **Shared and unique environmentality**. Shared and unique environmentality of liability to having a tattoo by age and 95% confidence intervals.
